# A frequency reconfigurable dipole antenna with solid-state plasma in silicon

**DOI:** 10.1038/s41598-018-33278-1

**Published:** 2018-10-09

**Authors:** Da-Jin Kim, Eon-Seok Jo, Young-Kyun Cho, Jae Hur, Choong-Ki Kim, Cheol Ho Kim, Bonghyuk Park, Dongho Kim, Yang-Kyu Choi

**Affiliations:** 1 School of Electrical Engineering, Korea Advanced Institute of Science and Technology, (KAIST) 291 Daehak-ro, Yuseong-gu, Daejeon 34141 Republic of Korea; 2 Department of Electrical Engineering, Sejong University, 209 Neungdong-ro, Seoul, 05006 Republic of Korea; 3 Giga-Communication Future Technology Research Group, Electronics and Telecommunications Research Institute, 218 Gajeong-ro, Yuseong-gu, Daejeon 34129 Republic of Korea

**Keywords:** Electrical and electronic engineering, Electronic devices

## Abstract

A frequency reconfigurable dipole antenna based on a silicon radiator is presented. The silicon radiator is activated with the aid of highly dense solid-state plasma by injecting carriers into the intrinsic region of p-i-n diodes. The fabrication and design guideline of the reconfigurable dipole antenna with this plasma radiator are described. When the plasma radiator is activated or deactivated, the length of the dipole arm changes, which means that the operating frequency of the dipole antenna is reconfigurable. When all the channels in the plasma radiator are activated, the operating frequency is tuned from 6.3 GHz to 4.9 GHz. The measured tunable bandwidth of our fabricated dipole antenna is approximately 31%, which is a practical value in comparison to conventional frequency reconfigurable antennas whose tunable bandwidth is in a range from 20% to 50%. To further support the validity of our results, we provide the well-matched simulation results from an antenna simulation. These results demonstrate that silicon with its commercial technology, which has not attracted attention in comparison to a metal antennas, is a promising tunable material for a frequency reconfigurable antenna. This plasma-based reconfigurable antenna has great potential for use in the dynamic communication environment.

## Introduction

Wireless data communication is playing an important role in our daily life because it offers flexibility and mobility. To transmit and receive the data among devices in various communication environments, a reconfigurable antenna that can fit various environments is needed^1^. In an overcrowded radio spectrum environment, a frequency reconfigurable antenna is an attractive solution to avoid interference among signals^2^. To communicate with various devices having different operating frequencies, a frequency reconfigurable antenna capable of adjusting its resonant frequency would be useful; therefore, the development of such an antenna has become a focus of research. Many types of frequency reconfigurable antennas using p-i-n diodes or varactor diodes have been studied^3–11^. In the case of p-i-n diodes, the operating frequency can be varied to meet predetermined demands, but continuous sweeping of the operating frequency is restricted^3–6^. In contrast, the varactor diode is a good solution for continuous frequency sweeping. However, the low self-resonant frequency of commercial varactor diodes restricts the tunable frequency range^7–11^. Moreover, a microelectromechanical systems (MEMS) switch, a reconfigurable feeding network, or tunable materials can also be adapted for a frequency reconfigurable antenna^12–14^. Among them, a frequency reconfigurable antenna using tunable materials has the potential to achieve continuous frequency sweep without low self-resonant frequency. For example, gas-phase plasma antennas have been widely studied because ionized gas plasma can substitute the metal part of the antenna due to its high conductivity when RF power is applied to the discharge tube, which can realize beam forming and alternation of the resonant frequency^15,16^. When the plasma is in an off-state, it has a stealth property due to its dielectric phase^17^. This characteristic has been primarily studied for military use, but it lacks practicality because the device for forming a gas-phase plasma has the disadvantages of bulky size, high power consumption, and difficulty for mass production.

In 2003, a feasibility study was reported in which the size and power consumption of a gas-phase antenna were reduced by forming plasma in a solid-state^18^. This antenna is called a solid-state plasma antenna, known as a plasma silicon antenna, because carriers are injected into solid-state silicon to form plasma with high conductivity. The concept of the plasma silicon antenna could replace a metal part of the antenna through the use of p-i-n diodes (a diode with a wide intrinsic (i) semiconductor region between a p-type doped region (p) and a n-type doped region (n)) in an on-state. In the on-state, the carriers are injected from the n-type and p-type heavily doped region to the intrinsic region by applying forward voltage between the p-type and n-type regions^19^.

The technique of controlling the carrier concentration in silicon is very common, and a typical use of this technique in a frequency reconfigurable antenna is to modulate the antenna characteristic by adopting varactor capable of adjusting the capacitance by DC biasing. The concepts of the plasma silicon antenna and the varactor are similar in terms of controlling carriers in silicon by DC biasing. However, the plasma silicon antenna must control the carriers in a much larger area to replace metal. When a solid-state plasma is formed in a large area, the temperature increases by Joule heat. Moreover, it is difficult to design an antenna due to embedded metallic elements, such as bias lines and gold wires, for applying DC bias. We overcame these problems in previous studies and showed that silicon material can be used as a reflector for a Yagi–Uda antenna^20^. However, the success of the silicon reflector only implies that the silicon has enough conductivity to simply reflect an incoming wave. To make various types of reconfigurable antennas, it is necessary to test whether such a silicon antenna can be designed to use silicon as a radiator. For this purpose, the AC signal should be transferred from the driven element to the silicon through integrated metal interconnections. To minimize the energy loss during the transfer, the silicon should have high conductivity. Moreover, the antenna should properly be designed to ensure impedance matching between the metal lines and the silicon. Because of the conductivity problem, the issue of impedance matching, and high temperature, no researchers have reported the measured antenna gain of a reconfigurable antenna using silicon as a radiator. Although previous studies have shown only the antenna input reflection coefficient (S_11_) from a monopole antenna, its performance was poor^21^. In this work, a half-wavelength dipole antenna using silicon as a radiator was fabricated, and the antenna gain was measured for the first time. We selected the dipole antenna because it is one of the most popular antennas due to its simple structure with various applications^22^. To achieve frequency reconfigurability, the dipole arms are made of a silicon p-i-n diode array. Consequently, we demonstrate a frequency reconfigurable dipole antenna, whose operating frequency can be changed by turning on or off each segment of p-i-n diodes connected in series with each other. The measured tunable bandwidth of the fabricated dipole antenna is approximately 31%, which is a practical value in comparison to conventional frequency reconfigurable antennas whose tunable bandwidth is in a range from 20% to 50%^3–10^. To the best of our knowledge, this is the first report of the measured gain of a frequency reconfigurable antenna whose radiator is made of silicon. To further support the validity of our result, we provide the well-matched simulation results obtained from an antenna simulation (CST software)^23^.

## Results and Discussion

**Figure 1:**
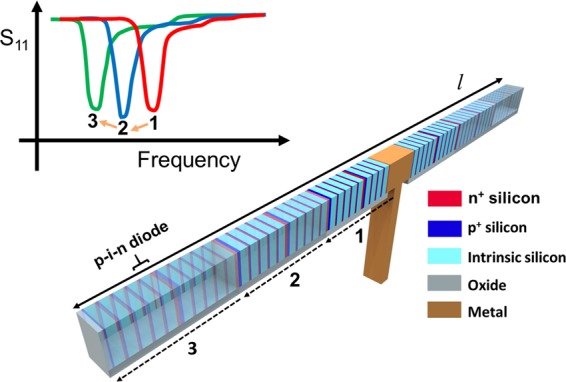
Operational principle of the proposed frequency reconfigurable dipole antenna.

The operational principle of the proposed reconfigurable dipole antenna is conceptually illustrated in Fig. 1. The operating frequency of the dipole antenna is reconfigurable by changing the length of the dipole arms. As illustrated in Fig. 1, shorter dipole arms result in a higher resonant frequency, which is determined by *f*_*r*_ = *c*/*λ*_*r*_ = *c*/2 *l* (*f*_*r*_: resonant frequency, *c*: speed of light, *λ*_*r*_: resonant wavelength, *l*: length of the dipole arm)^22^.

**Figure 2:**
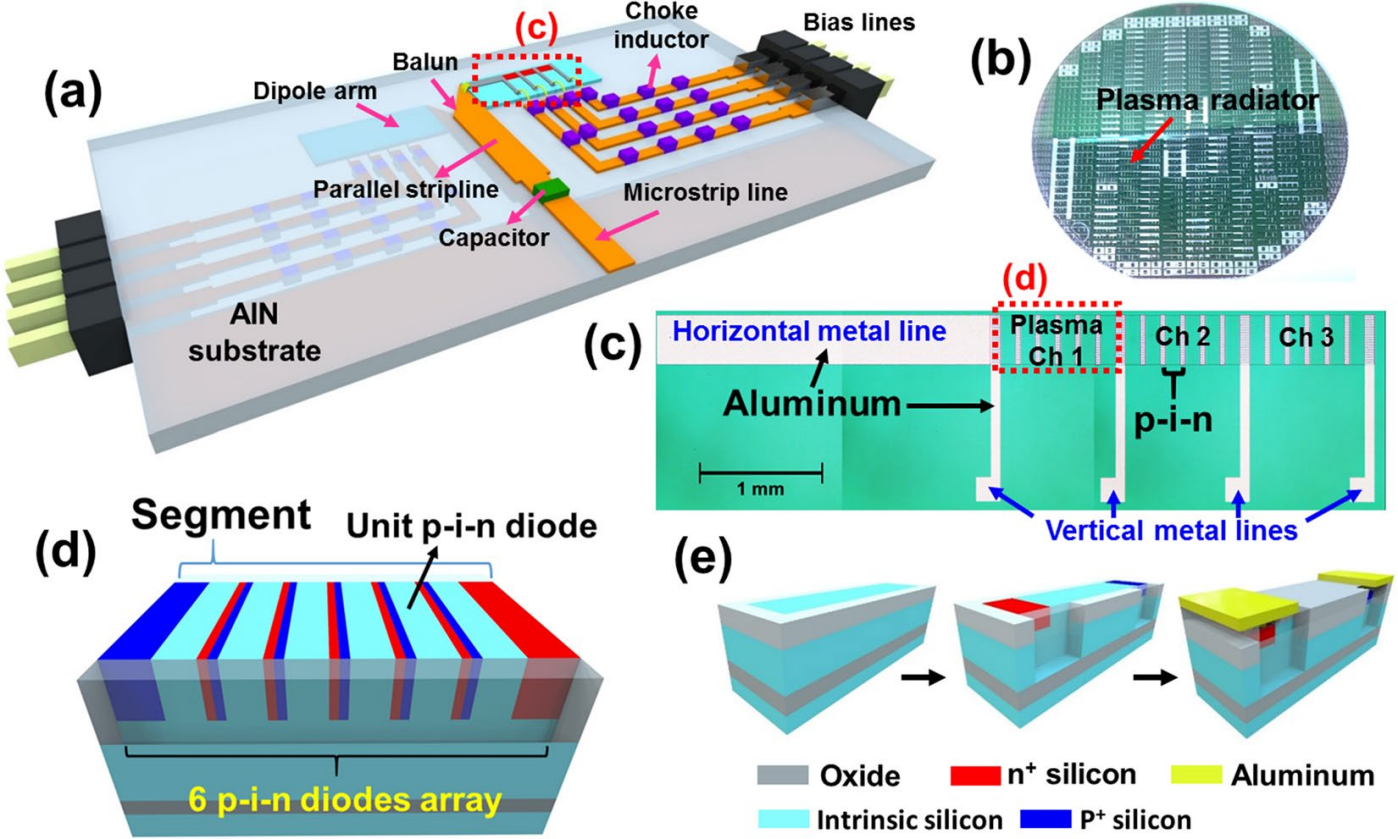
(**a**) Overall configuration of the proposed frequency reconfigurable dipole antenna. Black box with dotted line indicates the plasma radiator. (**b**) Optical photograph of the fabricated silicon wafer with a plasma radiator. (**c**) Optical photograph of the fabricated plasma radiator. Close-up view of the black box with dotted line. Red box with dotted line indicates the plasma channel. (**d**) Structure of the segment composed of serially connected p-i-n diodes. Metal lines are intentionally not drawn here. One segment makes a single plasma channel. (**e**) Fabrication flow of the unit p-i-n diode.

In this work, the solid-state plasma in silicon was used as a reconfigurable radiator by changing the length of the dipole arm, which is controllable by activating or deactivating each segment of the plasma radiator. The overall geometry of the proposed frequency reconfigurable dipole antenna is shown in Fig. 2(a). It consists of a microstrip line, a parallel stripline, and bias lines. They are patterned on both sides of a commercial AlN substrate with high thermal conductivity^24^ (*ε*_*r*_ = 8.56, *tan δ* = 0.0003, and thermal conductivity = 160 W/mK). The reconfigurable plasma radiator is located at the end of the parallel stripline, and it is connected to the bias lines by gold wires, which are required to activate the plasma radiator by supplying an external DC bias voltage.

First, the 50 Ω microstrip line is fed with an RF signal through a commercial coaxial connector. Next, the parallel stripline with a linearly tapered balun structure transmits the signal smoothly to the reconfigurable plasma radiator. For good impedance matching, the balun is optimized to have a tapered angle of *θ*_*t*_ = 57°. Also, the distance between the plasma radiator and the ground plane is set to about *λ*/4 (*λ*: wavelength of 4.7 GHz) to obtain a high antenna gain at 4.7 GHz.

We used a capacitor and inductors, which are needed to block DC power and RF signal, respectively. Specifically, the capacitor on the microstrip line couples an RF signal and blocks DC power. On the contrary, the choke inductors linked with the bias lines couple DC power and block RF signals. It is easy to preclude RF signals from flowing through the DC bias lines by greatly increasing the line impedance of the bias lines. However, in the case of a general inductor, high line impedance makes the bias lines very narrow, which cannot deal with a heating problem. Thus, we used choke inductors as an alternative.

In detail, the reconfigurable plasma radiator is composed of a horizontal metal line, vertical metal lines, and three plasma channels as shown in Fig. 2(c). Each dipole arm consists of the plasma channels and a horizontal metal line connected to the balun structure. The four narrow vertical metal lines are for DC bias. Since there are three plasma channels, the proposed antenna can be operated with four different frequencies according to the states of the plasma channels: all channels OFF, channel 1 ON, channels 1&2 ON, and all channels ON.

Unlike the conventional dipole antenna, the radiator of the proposed dipole antenna was fabricated using commercial silicon technology. In this silicon radiator, the conductivity of the silicon (*σ*_*si*_) should be as high as possible, while the wave launched from the driven element is radiated. In this work, a simple p-i-n diode array structure was selected to achieve a high *σ*_*si*_. The numbers of electrons and holes, which are proportional to *σ*_*si*_, can be modulated by applying forward voltage between the p-type and n-type regions. Holes are injected from the p-type heavily doped region to the intrinsic region, while electrons are injected from the n-type heavily doped region to the intrinsic region. As shown in Fig. 2(d), one segment is composed of serially connected p-i-n didoes, and several segments connected in parallel form the plasma radiator. Each segment is individually controlled by the bias lines and forms the plasma channel as shown in Fig. 2(a,c). The fabrication process of the unit p-i-n diodes is described in Fig. 2(e). First, to make the channel area, local oxidation of silicon (LOCOS) was utilized on an SOI wafer to isolate the active region from an adjacent dummy area. This isolation looks white in the perimeter on the top surface. Next, a highly n-type-doped (n^+^) junction was formed by phosphorus implantation with energy of 80 keV and a dose of 10^16^ cm^−2^. Similarly, the highly p-type-doped (p^+^) junction was formed by boron implantation with energy of 80 keV and a dose of 10^16^ cm^−2^. Afterward, a passivation layer of tetraethyl orthosilicate (TEOS) was deposited by low-pressure chemical vapor deposition (LPCVD) to protect the channel region from the environment. TEOS was also used to make a via hole for contact between the heavily doped region and the metal. Next, aluminum was deposited by sputtering and patterned according to the antenna design. Finally, forming gas annealing (FGA) was conducted to passivate the interface between the TEOS and top silicon^25,26^. An optical photograph of the completed silicon wafer with the plasma radiator is shown in Fig. 2(b). Over 200 of the plasma radiators were fabricated on 4-inch single wafer. Finally, the implanted bias lines on the substrate were connected to the metal pad of the radiator with gold wires.

**Figure 3:**
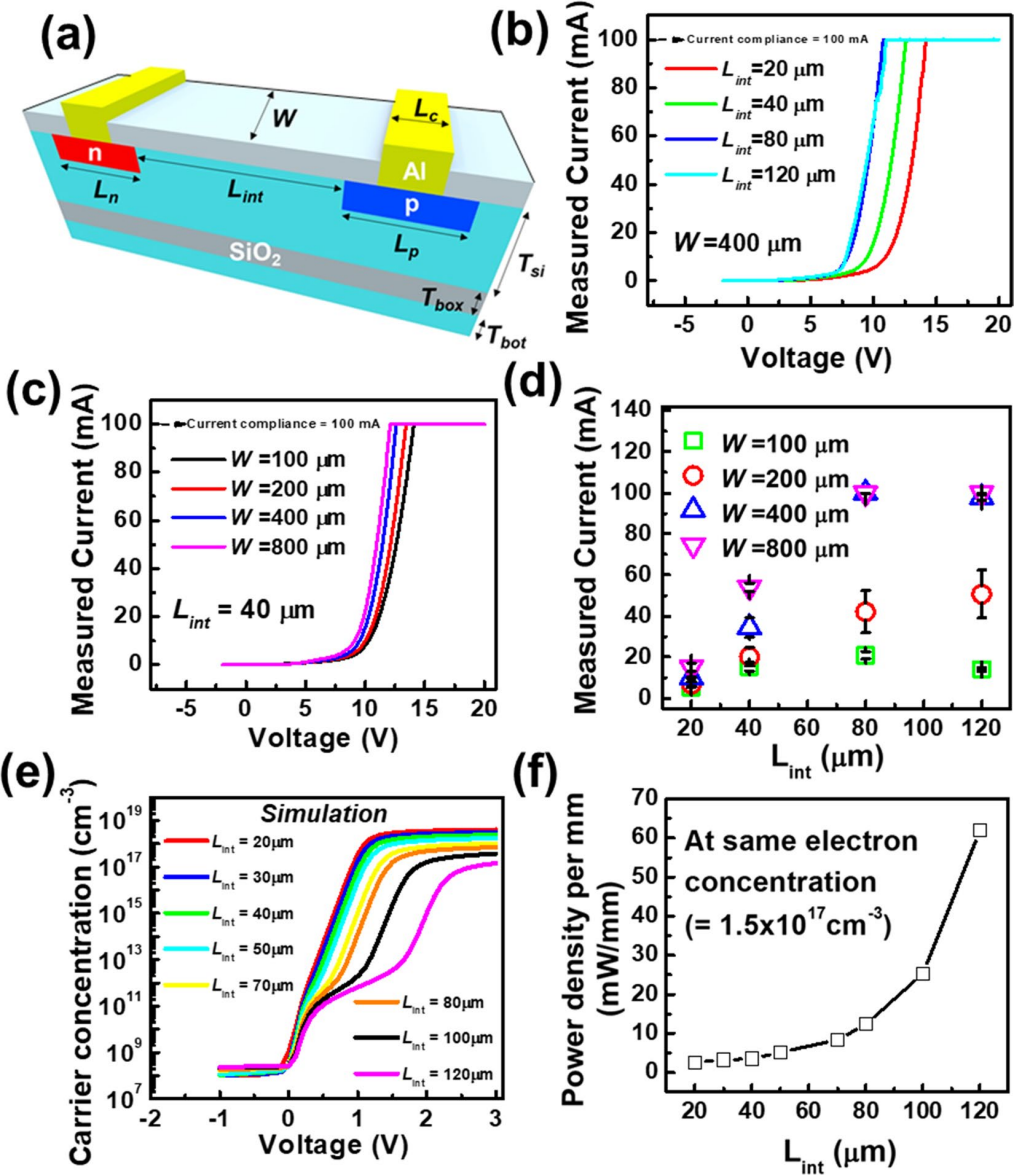
Measured and simulated electrical characteristics of the silicon radiator. (**a**) Geometry of the structural parameters in the unit p-i-n diode. *L*_*int*_ and *W* are variables, *L*_*c*_ is 10 μm, *T*_*box*_ is 1 μm, *T*_*si*_ is 50 μm, *T*_*bot*_ is 24 μm, and both *L*_*p*_ and *L*_*n*_ are 20 μm. (**b**) Measured I-V curves of the segment composed of serially connected p-i-n diodes according to *W*. (**c**) Measured I-V curves of the segment according to *L*_*int*_. (**d**) Measured current according to *W* and *L*_*int*_. (**e**) Simulated curves of the carrier concentration versus voltage according to *L*_*int*_. (**f**) Simulated curves of the power density versus *L*_*int*_ at the same electron concentration.

In the p-i-n diode of the silicon radiator, there are many device parameters to be optimized as shown in Fig. 3(a). However, the contact length (*L*_*c*_), the thickness of the buried oxide (*T*_*box*_), the length of the p-typed heavily doped region (*L*_*p*_), the thickness of the top silicon (*T*_*si*_), and the length of the n-type heavily doped region (*L*_*n*_) rarely affect the antenna characteristics when they are large enough. On the other hand, the intrinsic channel length (*L*_*int*_) and the channel width (*W)* of the p-i-n diodes significantly affect the antenna characteristics while the plasma radiator is activated. Therefore, the dimensions of the p-i-n diodes were set as follows: *L*_*int*_ and *W* are variables, *L*_*c*_ is 10 μm, *T*_*box*_ is 1 μm, *T*_*si*_ is 50 μm, *T*_*bot*_ is 24 μm, and both *L*_*p*_ and *L*_*n*_ are 20 μm. Figure 3(b) shows the forward current of a segment composed of six p-i-n diodes connected in series versus the applied voltage according to *L*_*int*_. The segment composed of p-i-n diodes with larger *L*_*int*_ exhibits a higher forward current because the number of p-i-n diodes is smaller, which means that the applied voltage of a single p-i-n diode is larger. In detail, to make a segment with a length of 960 μm (=6 × 160 μm), the required number of p-i-n diodes with *L*_*int*_ of 120 is six, including *L*_*p*_ of 20 μm and *L*_*n*_ of 20 μm. Similarly, the required number of p-i-n diodes with *L*_*int*_ of 20 μm is sixteen. Therefore, when the same voltage is applied to a single segment, a higher voltage is applied to the p-i-n diode with the length of 120 μm than that applied to the p-i-n diode with the length of 20 μm. In other words, a higher current flows in the channel length of 120 μm. As shown in Fig. 3(c), *W* of the p-i-n diodes was varied: 100 μm, 200 μm, 400 μm, and 800 μm. The forward current of the segment was linearly proportional to *W*. To increase the realized gain of the proposed antenna, a wider *W* was preferred (refer to Supplementary Information S1). Four types of *L*_*int*_ and four types of *W* were fabricated. The current with an applied voltage of 10 V for all the splits are plotted with an error bar in Fig. 3(d). For *W* of 100 μm, the current of the segment does not always increases as the *L*_*int*_ increases. It is supposed that when *W* is comparable to *L*_*int*_, the carriers are also captured at the sidewall of the intrinsic region where the interface between the silicon and the LOCOS oxide is formed. Although the segment with a larger *L*_*int*_ shows a higher current, that does not imply that the carrier concentration of the channel (intrinsic region) is also higher in the larger *L*_*int*_. Recombination time, diffusion length, distribution of the carrier concentration in the channel region, and length of the doped region should be considered when estimating the carrier concentration^27^. To extract the carrier concentration of each dimension of the segment, numerical simulations were carefully conducted with the aid of the SILVACO Atlas^28^. The average carrier concentration in the channel region versus applied voltage according to *L*_*int*_ is shown in Fig. 3(e). The p-i-n diode with a smaller *L*_*int*_ exhibits a larger carrier concentration at a given applied voltage. However, a smaller *L*_*int*_ requires a larger number of the p-i-n diodes to form a segment of the targeted length. For a fair comparison of carrier concentrations for different *L*_*int*_ values, the power density per mm should be considered including the length of the doped region. At the carrier concentration of 1.5 × 10^17^ cm^−3^, the segment with smaller *L*_*int*_ exhibits a smaller power density per mm as shown in Fig. 2(f). It is inferred that an *L*_*int*_ value less than 40 μm is preferred to achieve high a carrier concentration. Finally, the optimized device parameters were *W* of 400 μm and *L*_*int*_ of 40 μm.

**Figure 4:**
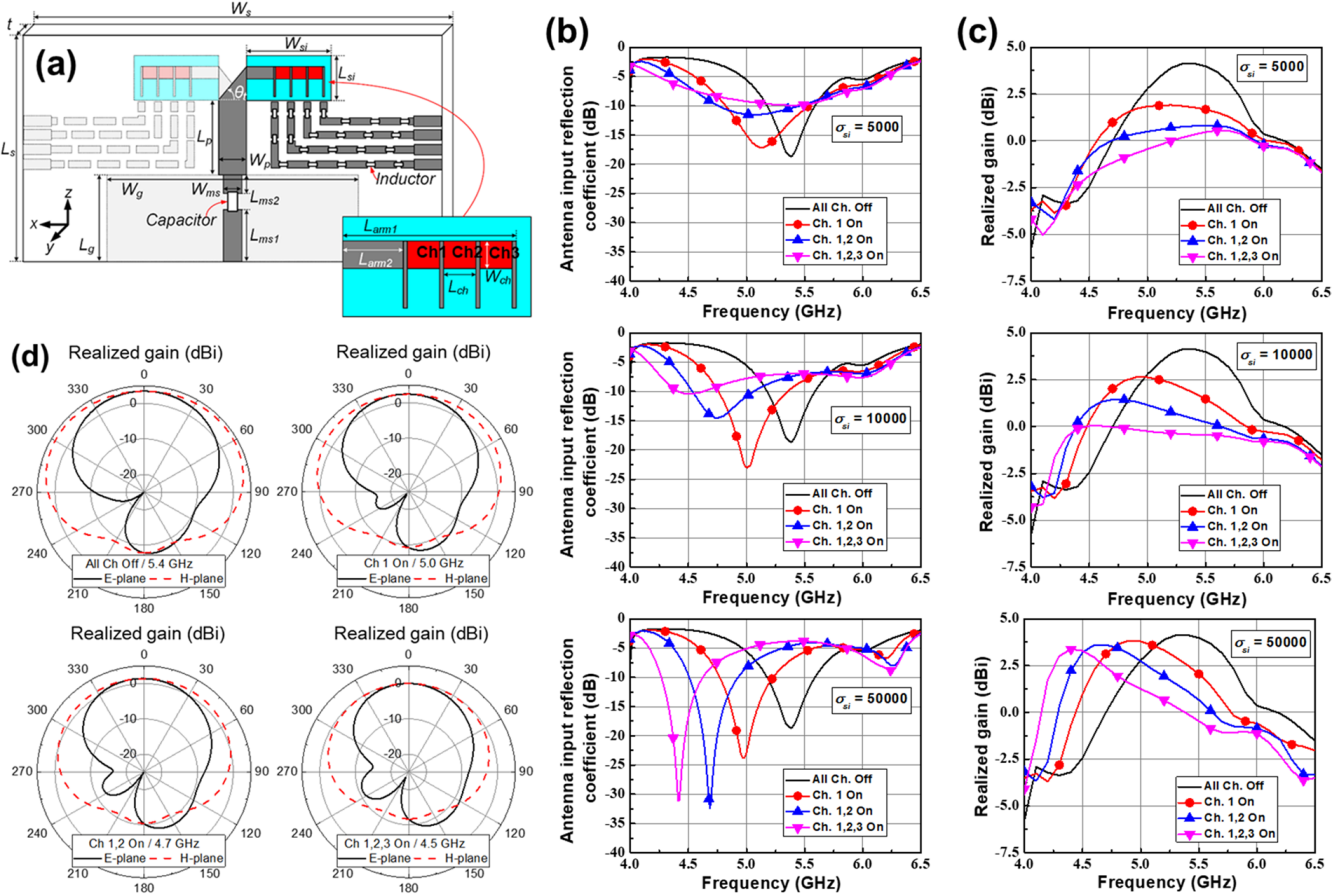
(**a**) Overall configuration of the simulated antenna. The dimensions of the antenna: *W*_*s*_ = 50 mm, *L*_*s*_ = 27 mm, *t* *=* 1 mm, *W*_*g*_ = 25 mm, *L*_*g*_ = 10 mm, *W*_*ms*_ = 1.09 mm, *L*_*ms1*_ = 8 mm, *L*_*ms2*_ = 1 mm, *W*_*p*_ = 1.72 mm, *L*_*p*_ = 10.38 mm, *W*_*si*_ = 7 mm, *L*_*si*_ = 3 mm, *L*_*arm1*_ = 5.8 mm, *L*_*arm2*_ = 2.72 mm, *L*_*ch*_ = 0.92 mm, and *W*_*ch*_ = 0.4 mm. (**b**) Simulated antenna input reflection coefficient according to *σ*_*si*_. (**c**) Simulated maximum realized gain according to *σ*_*si*_. (**d**) Simulated 2-D radiation patterns at each operating frequency.

Figure 4 shows the simulated antenna performance as a function of the number of activated plasma channels and the electrical conductivity of the plasma channels. First, the dimensions of the antenna were set as follows: *W*_*s*_ (width of the AIN substrate) = 50 mm, *L*_*s*_ (length of the AIN substrate) = 27 mm, *t* (thickness of the AIN substrate) = 1 mm, *W*_*g*_ (width of the partial ground plane) = 25 mm, *L*_*g*_ (length of the partial ground plane) = 10 mm, *W*_*ms*_ (width of the microstrip line) = 1.09 mm, *L*_*ms1*_ (length of the microstrip line under the blocking capacitor) = 8 mm, *L*_*ms2*_ (length of the microstrip line above the blocking capacitor) = 1 mm, *W*_*p*_ (width of the parallel microstrip line) = 1.72 mm, *L*_*p*_ (length of the parallel microstrip line) = 10.38 mm, *W*_*si*_ (width of the silicon radiator) = 7 mm, *L*_*si*_ (length of the silicon radiator) = 3 mm, *L*_*arm1*_ (length of the overall plasma dipole arm) = 5.8 mm, *L*_*arm2*_ (length of the aluminum dipole arm) = 2.72 mm, *L*_*ch*_ (length of the plasma channel) = 0.92 mm, and *W*_*ch*_ (width of the plasma channel) = 0.4 mm. From Fig. 4(b), we can reconfigure the operating frequencies by changing the number of activated plasma channels. In addition, the realized gain can also be tuned along with the reconfigured operating frequencies in Fig. 4(b). This means that the plasma channels radiate RF signals very well. In Fig. 4(b,c), it is worthwhile to focus on the effect of the electrical conductivity of the plasma channels on the behaviors of impedance matching and antenna gain. In the case of *σ*_*si*_ = 5,000, the operating frequency does not hop, and as the number of activated channels increases, the impedance matching property deteriorates. This means that *σ*_*si*_ of 5,000 S/m is not high enough to reconfigure the operation frequency band of the fabricated antenna. In contrast, in the cases of *σ*_*si*_ = 10,000 S/m and 50,000 S/m, the operating frequency and corresponding realized gain hop very well. The resonant frequency is shifted from 5.38 GHz to approximately 4.5 GHz according to the number of the turned-on channel. Moreover, though not shown here, the realized gain for *σ*_*si*_ = 50,000 S/m is similar to that of the same dipole antenna with metal arms. Figure 4(d) shows radiation patterns on the E- and H-planes for the number of activated plasma channels at each resonant frequency, while *σ*_*si*_ is set to 10,000 S/m. For easier understanding of radiation patterns of the proposed antenna, 3-dimensional (3D) radiation pattern of the proposed antenna is simulated (refer to Supplementary Information S2). Through the graphs, we can confirm the proposed antenna radiates well in the positive *z*-direction.

**Figure 5:**
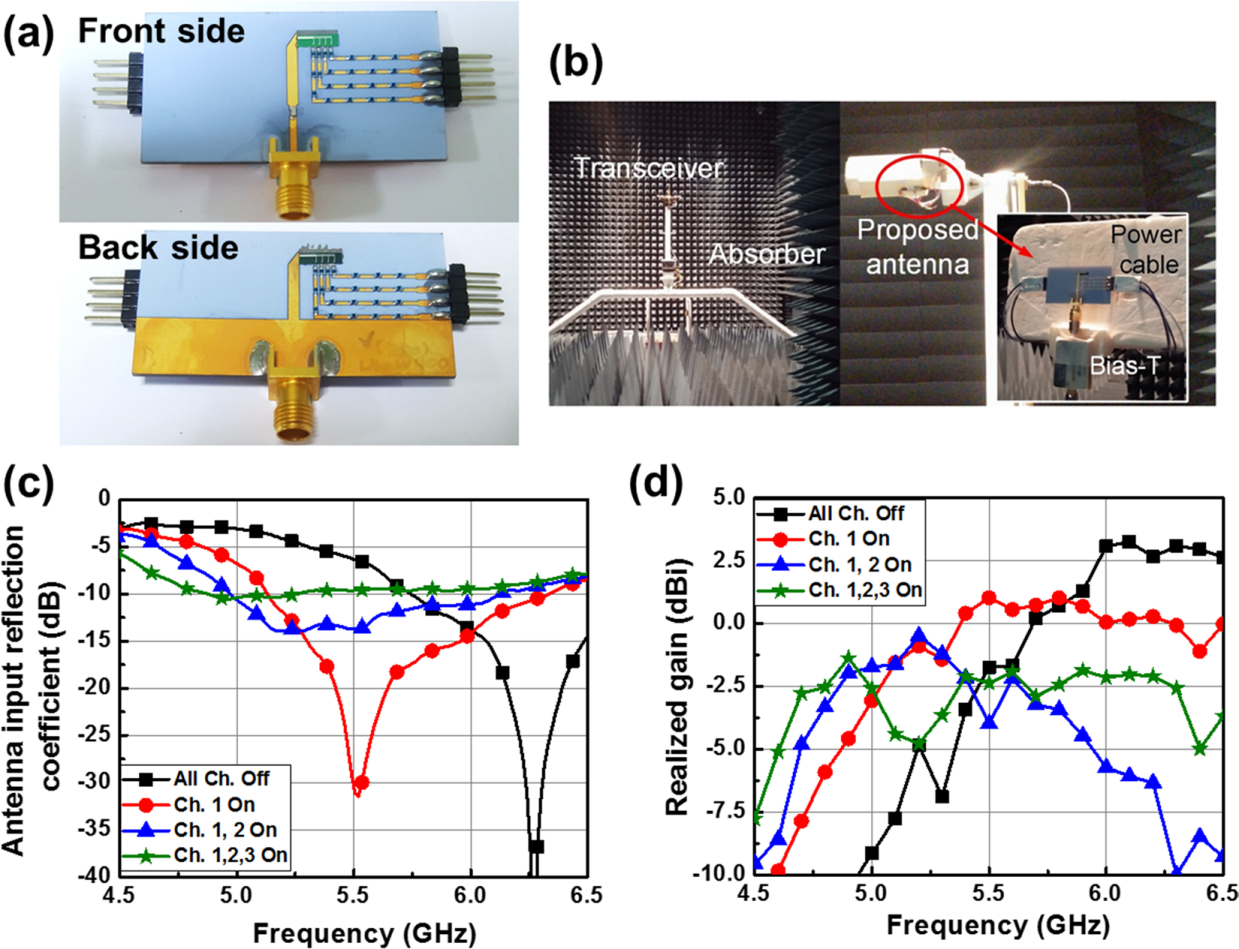
(**a**) Optical photograph of the fabricated dipole antenna. (**b**) Optical photograph of the fully anechoic chamber with the fabricated dipole antenna on a commercial Rohacell holder (**c**) Measured antenna input reflection coefficient. (**d**) Measured realized gain.

Figure 5(a) shows a photograph of the fabricated antenna with reconfigurable silicon dipole arms. Here, 12 V lithium battery is used to activate plasma channels. For activating a single plasma channel, approximately 1 W is needed. In other words, to activate all plasma channels, 6 W is required because each arm has three plasma channels. The experimental set-up in a fully anechoic chamber to measure the antenna radiation characteristics is presented in Fig. 5(b). As seen in Fig. 5(c), the measured operating frequency tunes from 6.3 GHz to 4.9 GHz as the number of activated plasma channels increases. Additionally, the antenna has a peak realized gain at each operating frequency as shown in Fig. 5(d), which means that the plasma channels radiates RF signals very well. Although the experimental results show a 10% frequency shift compared with the simulation result, the behaviors of the antenna input reflection coefficient and realized gain are very similar to the simulated results obtained for *σ*_*si*_ = 10,000 S/m. The difference between the simulated and measured results could be attributed to the temperature variation of the antenna substrate during the measurement. The high forward bias should be applied to obtain high conductivity. It inevitably creates a large amount of heat in the diode owing to the self-heating effect (refer to Supplementary Information S3). Thus, the temperature of the antenna substrate is increased hence this may affect the properties of the material used for the antenna, e.g., the dielectric constant of the substrate and the conductivity of the plasma radiator. In addition, the radiation interference among metallic elements, such as the DC-bias lines, gold wires, and connector, may cause the difference between the simulated and measured results. Gold wires in particular have an unpredictable effect on the antenna property because the exact geometry of the wires is hard to be considered in a simulation.

In this study, a plasma radiator was fabricated by commercial silicon technology to realize a reconfigurable dipole antenna. The operating frequency tunes from 6.3 GHz to 4.9 GHz as the number of the activated plasma channels increases. The experimental results showing a peak realized gain at each operating frequency, confirms that the plasma channels radiate RF signals very well. Such high performance was achieved by structural optimization with the aid of a device and antenna simulator as well as the good heat dissipation of the AlN substrate. By comparing the simulated and measured data of the dipole antenna, it was estimated that *σ*_*si*_ in the on-state is approximately 10,000 S/m. This good tunability of silicon achieved by controlling the number of carriers in the intrinsic channel region by applying voltage is highly attractive for making various types of reconfigurable antennas.

## Methods

### Fabrication of the antenna

The silicon-on-insulator (SOI) wafer was prepared by a commercial wafer bonding technology. Formation of the p-i-n diodes on the SOI wafer was conducted through a standard complementary metal oxide semiconductor (CMOS) silicon technology. The reconfigurable dipole antenna was fabricated on the AlN substrate (Rn2 Technologies, Co.) with metal patterns made by industrial silk screen printing. (Y. TECH, Co.) By silk screen printing, a silver layer with a thickness of 8 μm was deposited and patterned on the AlN substrate. Solder resist was also deposited on the silver patterns. Electroless plating was conducted to deposit a nickel layer with a thickness of 3 μm and gold layer with a thickness of 0.1 to 0.2 μm. All interconnections were provided by a commercial company. (RUATECH Inc.)

### Characterization

All electrical measurements were carried out without any device encapsulation. Electrical measurements were done with the aid of an HP4156 semiconductor parameter analyzer under ambient conditions. The antenna input reflection coefficient (S_11_) was measured using a vector network analyzer ZNB40 (Rohde & Schwarz GmbH). The realized gain was measured by an antenna measurement system with a full anechoic chamber in the IoT center, Incheon, South Korea. The antenna measurement system includes a PNA network analyzer E8362B (Agilent Technologies, Inc.), a microwave system amplifier 83017A (Keysight Technologies, Inc.), and a power supply 87421A (Keysight Technologies, Inc.). The gain-transfer method was used.

## Electronic supplementary material

Supplementary Information

## Data Availability

The datasets generated during and/or analyzed during the current study are available from the corresponding author on reasonable request.
