# Author Correction: A frequency reconfigurable dipole antenna with solid-state plasma in silicon

**DOI:** 10.1038/s41598-019-43506-x

**Published:** 2019-05-03

**Authors:** Da-Jin Kim, Eon-Seok Jo, Young-Kyun Cho, Jae Hur, Choong-Ki Kim, Cheol Ho Kim, Bonghyuk Park, Dongho Kim, Yang-Kyu Choi

**Affiliations:** 1 School of Electrical Engineering, Korea Advanced Institute of Science and Technology, (KAIST) 291 Daehak-ro, Yuseong-gu, Daejeon 34141 Republic of Korea; 2 Department of Electrical Engineering, Sejong University, 209 Neungdong-ro, Seoul, 05006 Republic of Korea; 3 Giga-Communication Future Technology Research Group, Electronics and Telecommunications Research Institute, 218 Gajeong-ro, Yuseong-gu, Daejeon, 34129 Republic of Korea

Correction to: *Scientific Reports* 10.1038/s41598-018-33278-1, published online 09 October 2018

In the original version of this Article, Cheol Ho Kim was incorrectly affiliated with ‘Department of Electrical Engineering, Sejong University, 209 Neungdong-ro, Seoul, 05006, Republic of Korea’. The correct affiliation is listed below.

Giga-Communication Future Technology Research Group, Electronics and Telecommunications Research Institute, 218 Gajeong-ro, Yuseong-gu, Daejeon, 34129, Republic of Korea

This error has now been corrected in the PDF and HTML versions of the Article, and in the accompanying Supplementary Information.

