# Supplementary material for: A frequency reconfigurable dipole antenna with solid-state plasma in silicon
Source: Sci Rep. 2018 Oct 9;8:14996. doi: 10.1038/s41598-018-33278-1 (PMC6177396; doi:10.1038/s41598-018-33278-1)
Supplement: Supplementary file 1 — Supplementary Information [file 41598_2018_33278_MOESM1_ESM.pdf]

Supplementary Information

A frequency reconfigurable dipole antenna with solid-state plasma in silicon

Da-Jin Kim,[1†] Eon-Seok Jo,[2†] Young-Kyun Cho,[3†] Jae Hur,[1] Choong-Ki Kim,[1] Cheol Ho Kim,[2] Bonghyuk Park,[3] Dongho Kim,[2,a)] and Yang-Kyu Choi[1,a)]

† These authors equally contributed to this work

[1]School of Electrical Engineering, Korea Advanced Institute of Science and Technology, (KAIST) 291 Daehak-ro, Yuseong-gu, Daejeon 34141, Republic of Korea

[2]Deparment of Electrical Engineering, Sejong University, 209 Neungdong-ro, Seoul 05006, Republic of Korea

[3]Giga-Communication Future Technology Research Group, Electronics and Telecommunications Research Institute, 218 Gajeong-ro, Yuseong-gu, Daejeon 34129, Republic of Korea

a) Authors to whom correspondence should be addressed.

Email addresses: dongkim@sejong.ac.kr and ykchoi@ee.kaist.ac.kr

Antenna characteristics according to the width of the silicon plasma channel

Figure S1 shows the influence of the width of the silicon plasma channel (W_{ch}) on antenna characteristics, the antenna input reflection coefficient and realized gain, when all the channels are turned on or off. The electrical conductivity of the activated channel is assumed to be 50000 S/m. First, the antenna matching characteristic becomes improved as W_{ch} increases. In addition, a wider frequency tunable bandwidth is observed. Also, the maximum antenna gain increases as W_{ch} increases, which means the wider W_{ch} is preferred for enhanced antenna performance. However, W_{ch} of 0.8 mm causes critical self-heating problem. Therefore, the optimal value of W_{ch} is 0.4 mm in our experiment.

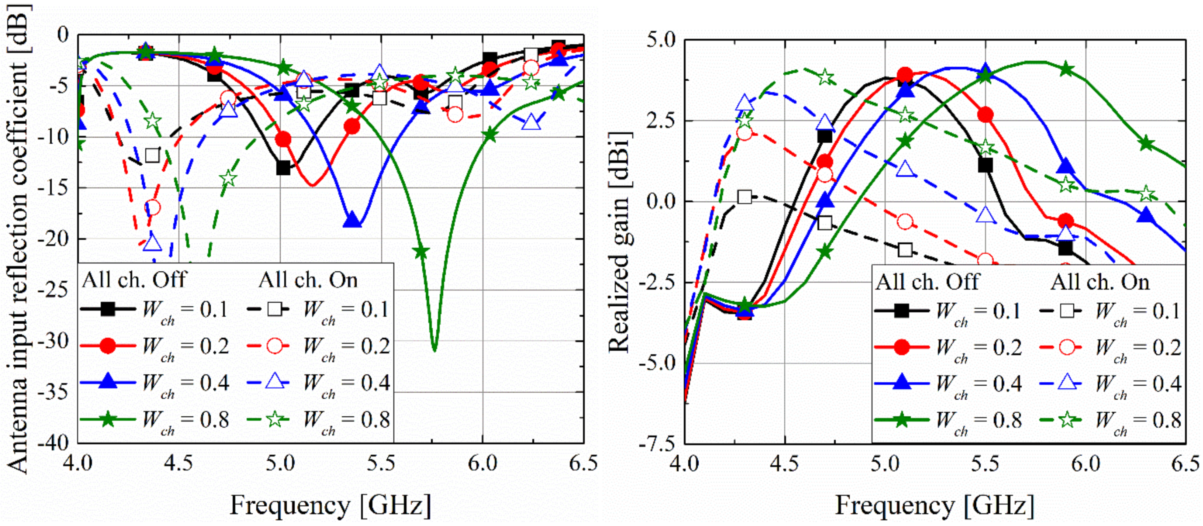

Figure S1. Antenna input reflection coefficient and maximum realized gain according to W_{ch}.

Simulated 3D radiation patterns of the proposed reconfigurable dipole antenna

As mentioned in the manuscript, we modeled and simulated the proposed antenna with the aid of CST Microwave Studio. Top and bottom view of the 3D modeled antenna are shown in Figure S2(a). Not only the geometrical structure but also silicon p-i-n diodes and RF components were included in the antenna simulation. Figure S2(b) shows 3D radiation patterns of the realized gain for the number of activated plasma channels at each resonant frequency. The electrical conductivity of the activated channel was set to 50,000 S/m. In all cases, the antenna radiates toward the positive *z*-direction and shows symmetric and wide beam width in H-plane.

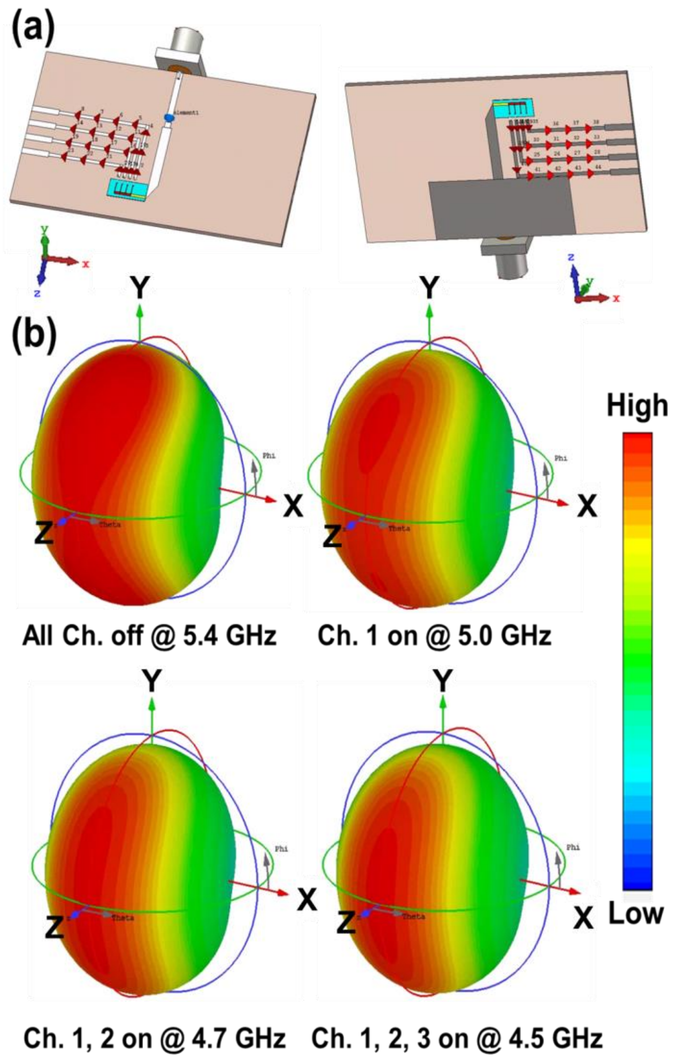

Figure S2. (a) Top and bottom view of the simulated antenna. (b) Simulated 3D radiation patterns at each operating frequency.

Temperature variation during antenna measurement

To monitor temperature variation during antenna measurement, we use a thermal imaging infrared camera (Flir Systems Inc.). The maximum temperature of the antenna substrate increases from 27.7°C to 90.1°C during the antenna operation. Due to high thermal conductivity of the AlN substrate, the maximum temperature is saturated approximately 90°C.

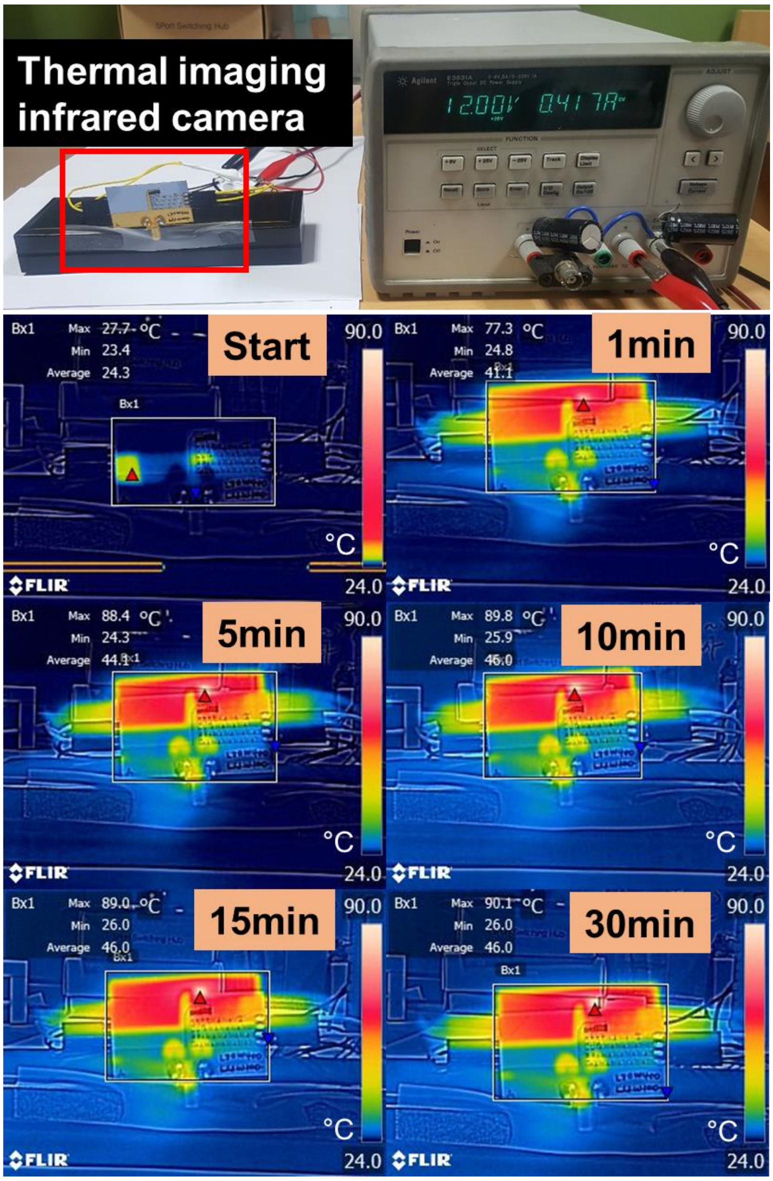

Figure S3. Measurement apparatus including DC power supply and the proposed antenna for temperature monitoring with a thermal imaging infrared camera. Thermal infrared images of the proposed antenna after 1 min, 5 min, 10 min, 15 min, 30 min operation compared with an initial image prior to enabling the antenna.
